# Severe Coronary Artery Disease in a Person Living with HIV

**DOI:** 10.3390/medicina57060595

**Published:** 2021-06-10

**Authors:** Mircea Bajdechi, Cosmin Mihai, Alexandru Scafa-Udriste, Ali Cherry, Diana Zamfir, Irina Dumitru, Roxana Cernat, Sorin Rugina

**Affiliations:** 1 Faculty of Medicine, University “Ovidius” of Constanta, 900470 Constanța, Romania; dumitrui@hotmail.com (I.D.); r.cernat@seanet.ro (R.C.); 2 Emergency Clinical Hospital of Bucharest, 014461 București, Romania; secretariat_FMed@univ-ovidius.ro (C.M.); spital@urgentafloreasca.ro (A.S.-U.); rectorat@umfcd.ro (A.C.); diana_zam74@yahoo.com (D.Z.); 3 4th Department, Cardio-Thoracic Pathology, University of Medicine and Pharmacy “Carol Davila” Bucharest, 050474 București, Romania

**Keywords:** coronaryrtery disease, acute coronary syndrome, coronary artery bypass grafting, percutaneous coronary intervention, HIV/SIDA, highly active antiretroviral therapy, drug–drug interactions

## Abstract

The pathophysiology of accelerated atherosclerosis in people living with Human Immunofediciency virus (HIV) is complex. Coronary artery disease (CAD) has become an important cause of mortality in these patients. They often have atypical symptoms, leading to frequently missed diagnoses. We report a case of a 51-year-old male undergoing antiretroviral therapy who was admitted for acute coronary syndrome. He had severe coronary artery disease that involved difficult management.

## 1. Introduction

Human immunodeficiency virus (HIV)-infected patients live longer now due to highly active antiretroviral therapy (HAART). Due to their increased life expectancy, they have an increased cardiovascular risk caused by the HIV itself and the specific therapy [1]. Cardiovascular deaths accounted for 6.5% [2] of all deaths in a large study of people living with HIV in Europe, 8% of such deaths in France [3], and 15% of deaths in a USA HIV outpatient study [4]. Coronary artery disease (CAD) has become an important cause of mortality in people living with HIV (PLHIV). The pathophysiology of accelerated atherosclerosis in PLHIV is complex. Antiretroviral therapy, inflammation, HIV-associated comorbidities such as dyslipidemia, drug abuse, opportunistic infections, lifestyle, and smoking are risk factors for HIV-associated atherosclerosis [5]. Often, drug–drug interactions are a real challenge in these patients.

## 2. Case Report

**Figure 1:**
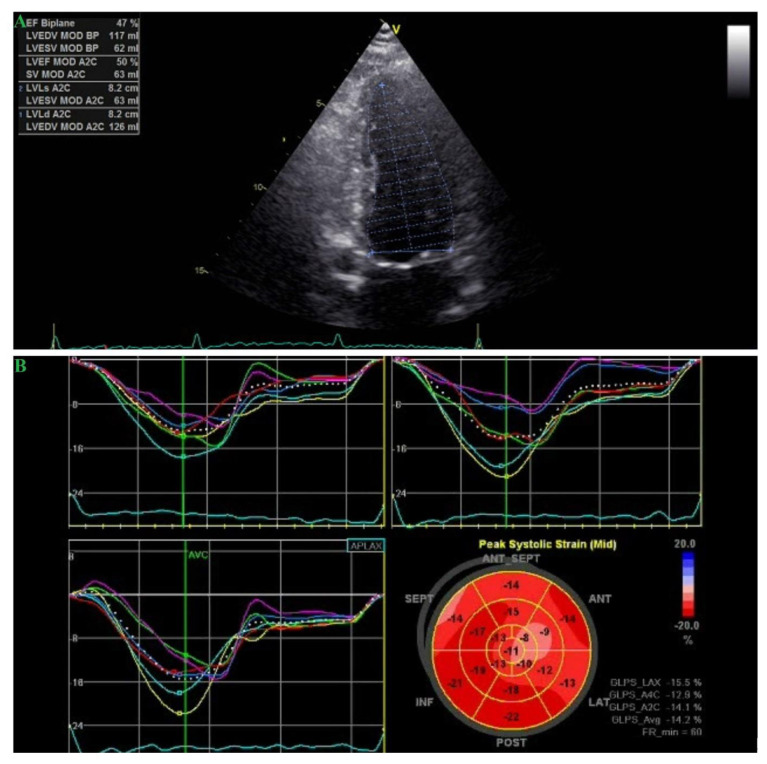
Transthoracic echocardiography: wall motion abnormalities in the left anterior descending artery territory with hypokinesia of the apical segments of the anterior and lateral wall and of the apex; borderline left ventricular systolic dysfunction (**A**) (EF(Ejection fraction) Biplane = 47%); (**B**) reduced peak systolic strain (Mid)—global longitudinal peak strain average (GLPS_Avg) 14.2%.

A 51-year-old Caucasian man, having lived for 20 years with HIV and undergone antiretroviral therapy (ART) including nucleoside reverse transcriptase inhibitors (NRTIs, lamivudine and zidovudine) and protease inhibitors (PIs—lopinavir and ritonavir) for more than 10 years, presented to the on-call room for atypical chest pain. The patient was a cigarette smoker (10 pack-years), of normal weight, and without a familial medical history of atherosclerosis. His physical examination and laboratory tests were unremarkable (normal lipid profile, undetectable viral load, and cluster of differentiation (CD4) = 507 cells/mm^3^). Resting transthoracic echocardiography showed a left ventricular cavity of normal size with mild systolic dysfunction—a left ventricular ejection fraction of 47% and wall motion abnormalities in the left anterior descending artery territory with hypokinesia of the apical segments of the anterior and lateral wall and of the apex, consistent also with the aspect of the polar map of global longitudinal strain (Figure 1). Furthermore, cardiac ultrasound revealed mild mitral regurgitation with a slightly enlarged left atrium (left atrium volume = 38 mL/m^2^) and low probability of pulmonary hypertension. The patient was admitted to hospital due to a high-risk cardiovascular profile and wall motion abnormalities, even though he was not presenting with typical angina.

**Figure 2:**
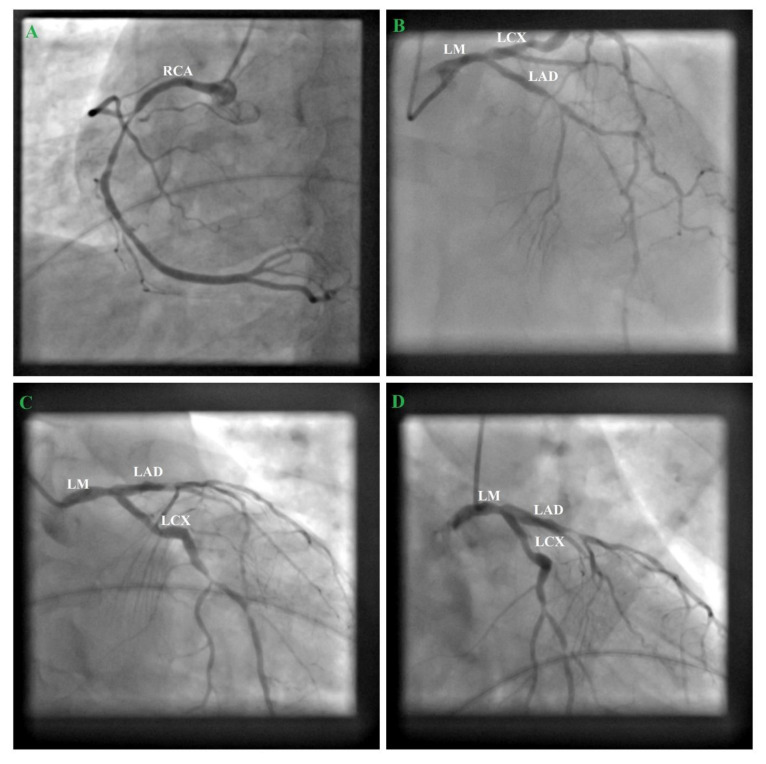
Coronary angiography. (**A**) Cranial projection of the right coronary artery (RCA) showing 90% stenosis in the mid segment of the RCA; (**B**) Cranial projection of the left coronary artery showing 90% stenosis in the proximal segment, a significant short obstructive lesion in the mid segment, and 60–70% stenosis in the distal segment of the left anterior descending artery (LAD); (**C**,**D**) Caudal projection of the left coronary artery showing a multiple 50–60% stenosis in the three segments of the left circumflex artery (LCX), 60–70% stenosis in the proximal segment of obtuse marginal 1 (OM1), and 50% stenosis in the distal segment of left main (LM).

Afterwards, the patient proceeded to coronary angiography, which revealed a left main coronary artery lesion and extensive triple coronary disease with extensive, diffuse atherosclerotic involvement of proximal and mid segments of all three coronaries and chronic LAD occlusion (Figure 2). In order to decide between CABG (coronary artery bypass graft) and PCI (percutaneous coronary intervention), we used the SYNTAX Score II, which showed a higher 4-year mortality for PCI (4.6% vs. 2.9% of CABG). To predict in-hospital mortality after cardiac surgery we calculated the EuroSCORE II, which showed a risk of in-hospital mortality of 1.45%. Therefore, we decided that surgery would be better suited for the patient, being able to provide a better outcome. He was transferred to a cardiac surgery clinic, where he underwent a successful coronary artery bypass graft using an isolated saphenous vein graft (on the left anterior descending artery, first diagonal artery, and right coronary artery), without immediate complications. We do not have a clearly stated reason as to why the surgeon chose venous grafts instead of arteries, but it might reflect personal preference.

He was discharged with medical therapy recommendations, which included aspirin, clopidogrel, rosuvastatin, and carvedilol. In agreement with the infectious disease doctor, due to drug–drug interactions (those receiving ritonavir are at risk of diminished clopidogrel response), we decided to stop the protease inhibitors so that an integrase inhibitor (raltegravir) could later be prescribed.

**Figure 3:**
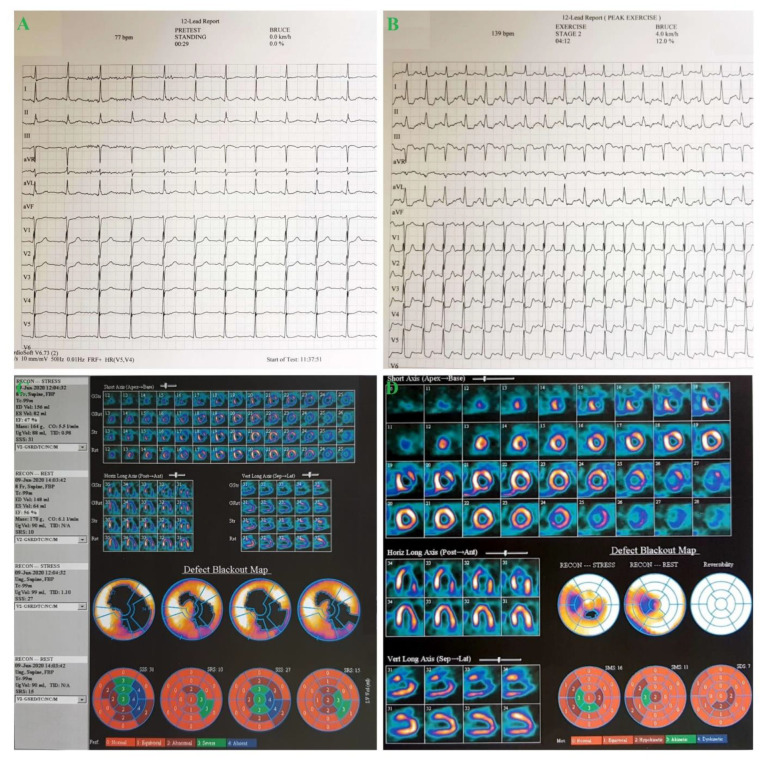
(**A**,**B**) Bruce protocol stress test—after 2 and a half minutes the patient complained of chest pain (8/10 intensity), and at the 4th minute (stage 2) the test was stopped because of the pain and electrocardiogram (ECG) findings: at 140 bpm, widespread horizontal ST depression, most prominent in DII, DIII, aVF and V4-V6 and ST elevation in aVR > 1 mm; (**C**,**D**) Myocardial perfusion scintigraphy stress induced mild left ventricular global hypokinesia and left ventricular systolic dysfunction (LVEF 45–47%).

Five months after the CABG, due to the recurrence of atypical angina pain, he underwent non-invasive ischemia tests. Therefore, he had a Bruce protocol stress test, which was positive (clinical and electrical) for ischemia (Figure 3), and further myocardial perfusion scintigraphy revealed inducible ischemia (Figure 3). The patient was sent to the cardiology department for further management.

**Figure 4:**
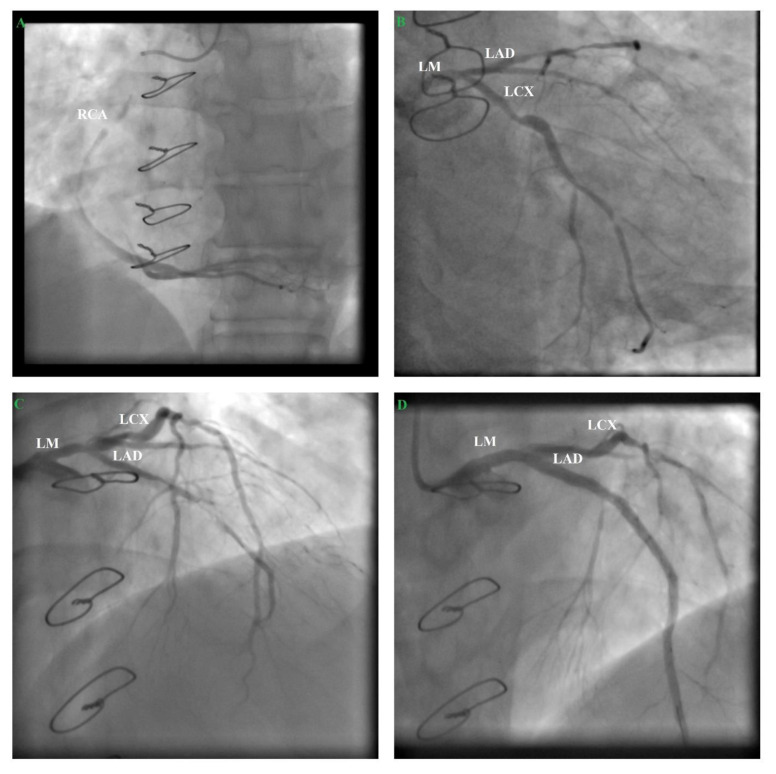
Percutaneous coronary intervention (PCI). (**A**) Cranial projection of the right coronary artery (RCA) showing 80% stenosis of the internal saphenous vein to the third segment of the right coronary artery; (**B**) Caudal projection of the left coronary artery showing a multiple 50–60% stenosis in the three segments of the left circumflex artery (LCX), 60–70% stenosis in the proximal segment of obtuse marginal 1 (OM1), and 50% stenosis in the distal segment of left main (LM); (**C**) Cranial projection of the left coronary showing occlusion of the internal saphenous vein to the first diagonal artery and 95% stenosis of the internal saphenous vein to LAD; (**D**) From distality to proximity—three DES on the whole LAD + one DES between the LM and LAD proximity, TIMI 3 flow.

Immediately, a coronary angiography was performed, which showed occlusion of the internal saphenous vein to the first diagonal artery, 95% stenosis of the internal saphenous vein to the left anterior descending artery, and 80% stenosis of the internal saphenous vein to the right coronary artery (Figure 4).

Taking into account the risks and benefits of percutaneous coronary intervention (PCI) versus a coronary artery bypass graft (CABG), EuroSCORE II 9.17% mortality risk, and Society of Thoracic Surgeons (STS) score 4.15% mortality risk, PCI with drug-eluting stents for revascularization was performed, with the patient’s consent. We decided to perform staged PCI revascularization. First of all, we implanted three drug-eluding stents (DESs) on the whole left anterior descending artery (LAD) and one DES between the left main artery distality and LAD proximity (Figure 4). After this procedure, all the coronary arteries had Thrombolysis in Myocardial Infarction (TIMI) 3 flow (Figure 4). He was discharged with ticagrelor instead of clopidogrel and a maximal atorvastatin dose instead of rosuvastatin.

**Figure 5:**
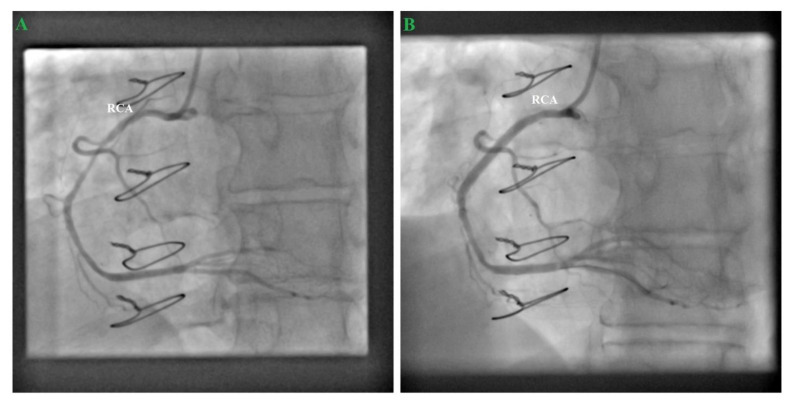
(**A**) Cranial projection of the right coronary artery (RCA) showing, at diagnosis, that the internal saphenous vein graft to the RCA was totally occluded this time around; (**B**) Drug-eluding stent angioplasty on the RCA mid segment, TIMI 3 flow.

One month later, the patient came for right coronary artery (RCA) elective angioplasty, so we performed drug-eluding stent angioplasty on the RCA mid segment, with good results. Interestingly, at diagnosis the internal saphenous vein graft to the right coronary artery was totally occluded this time around (Figure 5). At a 5-month follow-up, the patient had a good clinical status, without angina, with a good effort tolerance and improved quality of life.

## 3. Discussion

Although this patient did not have typical symptoms, taking into account his HIV infection treated with HAART, we considered him to be at high risk of having an acute coronary syndrome. Reduced Global Longitudinal Strain showed us significant ischemic changes and led to further angiography, which revealed severe coronary artery disease requiring coronary artery bypass grafting (CABG). Less than six months later, the patient needed repeat revascularization via percutaneous coronary intervention (PCI) of the native coronary arteries.

It is unknown whether people living with HIV (PLHIV) have a higher frequency of atypical symptoms such as silent ischemia that can be observed in chronic diseases (diabetes mellitus or chronic kidney disease) [6].

There are different data regarding the difference between the incidence rates of cardiac vascular disease in PLHIV and in HIV-negative individuals. PLHIV do not differ from the others in terms of risk factors and angiographic characteristics after a first episode of acute coronary syndrome, except for higher rates of illicit drug use and hypertriglyceridemia in PLHIV [7]. Recent data showed that the overall risk of major adverse cardiac and cerebrovascular events was not statistically significantly different between the two groups, but a higher risk of recurrent ischemic events and hospitalizations for heart failure after a first acute coronary syndrome in PLHIV was observed [8]. Older studies showed that the hospitalization rate for coronary heart disease was significantly higher in PLHIV compared to HIV-negative members [9,10].

The long-term prognosis after an acute coronary syndrome in people living with HIV is unclear [8], but it is certain that these patients have a worse cardiovascular risk profile.

The prevalence of left main disease is 6%, which is equivalent to that in HIV-uninfected persons [11,12]. CABG has been considered the gold-standard treatment of left main coronary disease lesions. Literature data on cardiac revascularization in PLHIV are still scarce. Coronary artery bypass grafting (CABG) is a feasible technique for revascularization in PLHIV. Boccara et al. compared 27 PLHIV and 54 controls without HIV infection undergoing CABG, and after 30 days, both groups had the same rates of major adverse cardiovascular events; however, at a median follow-up of 41 months, there was a higher rate of adverse events in the HIV-infected cohort versus controls, mostly because of the need for repeated revascularization using PCI of the native coronary arteries, and not of the grafts [13].

Antiretroviral-associated endothelial dysfunction has been described in many studies and may be associated with the increased incidence of cardiovascular diseases. Experimental studies demonstrated that in mice, antiretrovirals such as nucleoside reverse transcriptase inhibitors (NRTIs) or PIs induce endothelial injury after only a few days of treatment [14]. These antiretrovirals can exacerbate injury-induced vascular smooth muscle cell proliferation and induce neointimal hyperplasia, which is an important component of atherosclerosis. Furthermore, the HIV itself, through many possible pathways, is a recognized risk factor for atherosclerosis. The effects of the virus and of the antiretrovirals promoting endothelial dysfunction are additive. New studies on higher platelet reactivity could provide new insights into the higher risk of atherothrombosis in PLHIV and could lead to specific new therapies [8].

Drug–drug interactions also represent a problem in this case. The coadministration of clopidogrel or prasugrel and boosted regimens has been evaluated in studies. Prasugrel appears to remain an adequate antiplatelet agent in these patients and could be preferred to clopidogrel, regardless of the metabolic interaction and inhibition of its bioactivation pathways [15]. Unfortunately, in Romania, prasugrel is not available, so we had to choose ticagrelor, but coadministration of ticagrelor with strong inhibitors of Cytochrome P359 3A4 (CYP3A4) is contraindicated. Also, the combination of atorvastatin or rosuvastatin with PIs is not recommended. Thus, together with the infectious disease doctor and with the patient’s consent, the patient decided to change PIs with an integrase inhibitor (raltegravir).

The spectrum of myocardial disease in people living with HIV ranges from incidental asymptomatic findings to symptomatic disease [16]. Before the antiretroviral era, the main cardiac impairments in these patients were pericarditis, myocarditis, and endocarditis. Now, the prevalence of these diseases has dropped significantly. Literature data show low percentages of HIV infection in large groups of patients with endocarditis [17] or pericarditis [18].

The pathophysiology of accelerated atherosclerosis in these patients is complex, including traditional atherosclerotic risk factors with a higher prevalence, endothelial damage and dysfunction, hypercoagulability, local and systemic inflammation, altered immune responses, and metabolic disorders from antiretroviral therapy. Coronary artery disease (CAD) has become an important cause of mortality in people living with HIV. Cardiac ultrasonography is a useful tool in standard clinical practice and is able to detect left ventricular systolic dysfunction in patients with acute coronary syndromes [19].

Considering Framingham Risk scores and the atherosclerotic cardiovascular disease (ASCVD) risk algorithm, the cardiovascular disease risk in people living with HIV is underestimated [20].

## 4. Conclusions

People living with HIV have accelerated atherosclerosis. It can lead to severe coronary artery disease with unspecific symptoms. Non-invasive tests to detect myocardial ischemia, such as stress cardiac magnetic resonance, myocardial perfusion scintigraphy, and coronary CT angiography, should be studied for detecting early stage disease. Coronary artery bypass grafting using arteries is preferred due to the increased risk of venous occlusion. Percutaneous coronary intervention with drug-eluding stents remains an option, even though the stenting is often extensive and there is the risk of target lesion revascularization failure in the long term. Drug–drug interactions are a real problem in people living with HIV, and treatment must be continuous with a strong collaboration between the infectious disease specialist and the cardiologist.

## Data Availability

Not applicable.
